# ESPRIT-Like Two-Dimensional DOA Estimation for Monostatic MIMO Radar with Electromagnetic Vector Received Sensors under the Condition of Gain and Phase Uncertainties and Mutual Coupling

**DOI:** 10.3390/s17112457

**Published:** 2017-10-26

**Authors:** Dong Zhang, Yongshun Zhang, Guimei Zheng, Cunqian Feng, Jun Tang

**Affiliations:** 1 Air and Missile Defense College, Air Force Engineering University, Xi’an 710051, China; zysyjtc@163.com(Y.Z.); zheng-gm@163.com(G.Z.); dzdktougao@sina.cn(C.F.); 2 Department of Electronic Engineering, Tsinghua University, Beijing 100084, China; Tangj_ee@mail.tsinghua.edu.cn

**Keywords:** 2D-DOA, MIMO radar, electromagnetic vector received sensors, gain and phase uncertainties, mutual coupling

## Abstract

In this paper, we focus on the problem of two-dimensional direction of arrival (2D-DOA) estimation for monostatic MIMO Radar with electromagnetic vector received sensors (MIMO-EMVSs) under the condition of gain and phase uncertainties (GPU) and mutual coupling (MC). GPU would spoil the invariance property of the EMVSs in MIMO-EMVSs, thus the effective ESPRIT algorithm unable to be used directly. Then we put forward a C-SPD ESPRIT-like algorithm. It estimates the 2D-DOA and polarization station angle (PSA) based on the instrumental sensors method (ISM). The C-SPD ESPRIT-like algorithm can obtain good angle estimation accuracy without knowing the GPU. Furthermore, it can be applied to arbitrary array configuration and has low complexity for avoiding the angle searching procedure. When MC and GPU exist together between the elements of EMVSs, in order to make our algorithm feasible, we derive a class of separated electromagnetic vector receiver and give the S-SPD ESPRIT-like algorithm. It can solve the problem of GPU and MC efficiently. And the array configuration can be arbitrary. The effectiveness of our proposed algorithms is verified by the simulation result.

## 1. Introduction

Since its first appearance more than a decade ago, Multiple-Input Multiple-Output (MIMO) radar has become a research hotspot [1,2,3]. It makes use of multiple antennas to emit orthogonal waveforms and utilizes multiple antennas to receive the echoes which will bring unique advantages [4]. It also can emit correlated waveforms to achieve more flexible beam pattern designs [5]. There are many methods for beampattern design, such as the classic Capon beamforming method and its improved form [6,7,8]. For MIMO radar, we can realize the beampattern design through flexible waveform design [9]. For example, Ahmed et al. [10] propose a transmit beampattern design method based on one step unconstrained optimization, which avoids the design of waveform covariance matrix and reduces the complexity. The robust waveform covariance matrix design with minimum sidelobe level or minimum integrated sidelobe level is studied in [11] and it also gives a design method which has polynomial time complexity. There are two categories of MIMO radar. One is statistical MIMO radar whose array spacing is very large. It can achieve diversity gains and overcome the effect of target scintillation [12,13]. The other is colocated MIMO whose array elements are closely spaced [14,15,16]. This can improve the parameter estimation performance significantly. Angle estimation is very important in radar system [17], so in this paper, we pay attention to the 2D-DOA estimation of collocated monostatic MIMO radars.

There are many algorithms for direction of arrival (DOA) estimation, such as the Capon method [18], multiple signal classification (MUSIC) algorithm [19], and the estimation of signal parameter via rotational invariance technique (ESPRIT) algorithm [20]. In addition, the sparse signal reconstruction-based DOA estimation algorithm has become a hot research topic [21]. These algorithms can achieve high estimation accuracy and have been successfully applied to the DOA and direction of departure (DOD) estimation in MIMO radar [22,23,24]. Among them, the ESPRIT algorithm has received extensive attention because of its high computational efficiency.

Polarization sensitive array signal processing is a new bench of array signal processing [25] that has received a lot of attention recently [26,27]. A polarization array is an array consisting of multiple polarization- sensitive units which are spatially distributed in a certain structure and are not identical in polarization [28]. Unlike conventional scalar sensor arrays, polarization-sensitive arrays can acquire polarization direction information, that is, they have polarization diversity capability. This outstanding feature provides a new and useful means for information acquisition and transmission [29].

The combination of a MIMO array and a polarization-sensitive array will bring much more performance improvement in parameter estimation [30]. Based on this, Jiang et al. [31] proposed polarimetric MIMO radar. Its transmitting array consists of scalar antennas and its receiving array is composed of cross-dipole vector antennas. Then it utilizes ESPRIT algorithm to estimate DOD, DOA and polarization station angle (PSA). Gu et al. [30] used a six-component electromagnetic vector sensor (EMVS) to increase the information of a single vector antenna. As we all know, the spatial electromagnetic wave signal is a vector signal. The complete electric and magnetic information of a vector signal is a complex vector with six dimensions. Most of the existing radar systems use a single polarization scalar array to obtain only one-dimensional electromagnetic wave signal information. The electromagnetic vector sensor can get all or at least more than one dimension information of space electromagnetic wave signals. For example, Jiang et al. [31] adopted cross-dipole antennas. Therefore, the vector antennas only obtain two-component electric-field of source. Gu et al. [30] adopted a six-component electromagnetic vector-sensor which uses three orthogonal electric-dipoles and magnetic-loops to measure the three electric-field- and three magnetic-field parameters of the incident signals, respectively. Thus the EMVS can get complete electromagnetic vector information. The EMVS is a new type of single input multiple output antenna which receiving close attention from academia and industry. Because it uses more information, we can get more advantages in DOA estimation. For example, we can use the relationship between these electromagnetic components to estimate DOA. Besides, we can combine it with the ESPRIT results to improve the estimation accuracy of DOA. Then, Zheng et al. [26] extended EMVS to the MIMO radar. It uses the rotational invariance of the spatial-polarimetric domain (SPD) and proposes a corresponding ESPRIT-like method to estimate the 2D-DOA and PSA in monostatic MIMO radar. It is suitable for arbitrary array configurations.

However, all the above methods are based on the hypothesis that there are no gain and phase uncertainties (GPU) and the mutual coupling (MC) of EMVS components is also not considered. There are too many uncertainties in the actual production of the antenna, the amplitude and the phase gain of different array elements cannot be exactly the same, so amplitude and phase errors and mutual coupling are very common in practical situations. When the element spacing is relatively close, the mutual coupling between the elements is unavoidable, so in practice, the array manifold is inevitably affected by mutual coupling and array gain or phase uncertainties. This results in significant distortion of the amplitude and phase of the signals received from the array, so the direct use of these methods will lead to a serious degradation of estimation accuracy or even failure. How to eliminate this effect is a meaningful task. There are many people researching the problem of GPU and MC. Wang et al. [32] solved the MC problem of a uniform linear array (ULA) based on subspace theory. In [33], a robust direction-of-arrival (DOA) estimation algorithm for quasi-stationary signals based on the Khatri-Rao (KR) subspace approach was put forward. It can solve the GPU problem of DOA estimation. Si et al. [34] tackled the GPU and MC problem for ULAs or uniform circular arrays (UCAs) by applying a group of auxiliary sensors. However, for the problem of GPU and MC in MIMO-EMVS, as far as we know, there are no corresponding references, so in this paper, we studied the GPU and MC problem of MIMO-EMVS.

Firstly, we propose a spatial-polarimetric domain ESPRIT-like algorithm based on centralized EMVS (C-SPD ESPRIT-like algorithm) for the estimation of 2D-DOA and PSA in MIMO-EMVSs under the condition of GPU. The EMVS adopts a centralized configuration. This algorithm can be applied to an arbitrary array configuration. Then, considering the mutual coupling of components of EMVS, we revise the C-SPD ESPRIT-like algorithm and apply it to a class of separated EMVSs. The GPU and MC problem are solved simultaneously. The proposed methods have low complexity for avoiding the angle searching operation and the array configuration can be arbitrary. And the effectiveness of proposed algorithms is verified by the simulation results.

The remainder of the paper is organized as follows: Section 2 is devoted to the introduction of the signal model of polarimetric MIMO radar. In Section 3, the two proposed methods for estimation of DOA under non-ideal conditions are described, and some discussions are presented. Section 4 gives a comparison of these methods. Simulation results are presented in Section 5 to evaluate the advantages of the proposed methods. At last, conclusions are drawn in Section 6.

## 2. Signal Model

**Figure 1:**
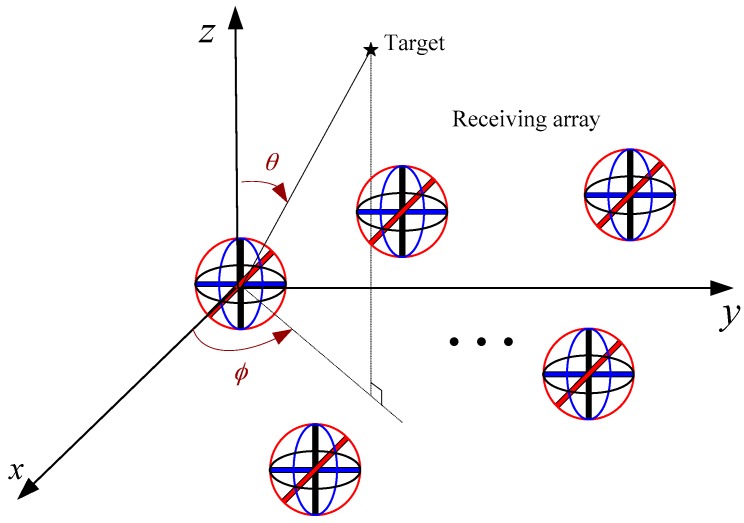
Monostatic MIMO radar with arbitrarily spaced centralized EMVSs.

Assuming there is a monostatic MIMO radar which hass *M* transmitters and *N* − 1 receivers. Each receiver is composed of a six-component centralized EMVS which is shown in Figure 1. As we all know, the spatial electromagnetic wave signal is a vector signal. The complete electric and magnetic information of a vector signal is the complex vector of six dimensions. This six dimensional vector is related to the DOA and polarization station angle of the target. The polarization station angle is composed of a polarization phase angle and the polarization phase difference. The EMVS consists of the phase centered coincidence of three orthogonal electric dipoles and three orthogonal magnetic rings that can get complete electromagnetic vector information, so each EMVS has six component outputs, representing the electric field and magnetic field components of the incident electromagnetic wave. In our model, the position of the *m*-th array element in the transmitting array is $\left(x_{tm},y_{tm},z_{tm}\right),m=1,2,\ldots ,M$ and the position of the *n*-th array element in the receiving array is $\left(x_{tn},y_{tn},z_{tn}\right),n=1,2,\ldots ,N-1$. There is no limitation of the array configuration of both the the transmitting array and the receiving array. That is to say they are arbitrary array configurations and later we will show that our algorithm has no requirement for a specific array configuration.

Assuming the transmitting waveforms are normalized orthogonal signals, i.e., $\left(1/\Gamma \right)\sum_{\tau =1}^{\Gamma }s\left(\tau \right)s^{H}\left(\tau \right)=I_{M}$, where $s\left(\tau \right)={\left[s_{1}\left(\tau \right),s_{2}\left(\tau \right),\ldots ,s_{M}\left(\tau \right)\right]}^{T}\in {\mathbb{C}}^{M\times 1}$ denotes M transmitted signals, τ=1,2,…,Γ is the sampling index in fast time. There are K far field uncorrelated point targets. The 2D-DOA of incident signals can be expressed as azimuth angle ϕ∈[0,2π) and elevation angle θ∈[0,π]. They are measured from the positive x-axis and positive z-axis, respectively. The PSA of targets are auxiliary polarization angle γ∈[0,π/2] and polarization phase difference η∈(−π,π], respectively. The received data can be expressed as follows:

(1) $$x\left(t,\tau \right)=\Lambda_{r}\left[A_{r}\left(\theta ,\phi \right)⊕A_{pol}\left(\theta ,\phi ,\gamma ,\eta \right)\right]\mathrm{diag}\left[b\left(t\right)\right]{\left[\Lambda_{t}A_{t}\left(\theta ,\phi \right)\right]}^{T}s\left(\tau \right)+w\left(t,\tau \right)$$

where t is the slow-time, i.e., snapshot. $\Lambda_{t}$ is a M×M complex diagonal matrix which represents the GPU of transmitting sensors. $\Lambda_{r}$ is a 6(N−1)×6(N−1) complex diagonal matrix which represents the GPU of receiving sensors. $A_{r}\left(\theta ,\phi \right)=[a_{r}(\theta_{1},\phi_{1}),\ldots ,a_{r}(\theta_{K},\phi_{K})]\in {\mathbb{C}}^{\left(N-1\right)\times K}$ is the receiving spatially steering matrix. $A_{t}\left(\theta ,\phi \right)=[a_{t}(\theta_{1},\phi_{1}),\ldots ,a_{t}(\theta_{K},\phi_{K})]\in {\mathbb{C}}^{M\times K}$ is the transmitting spatially steering matrix. $A_{pol}\left(\theta ,\phi \right)=[a_{pol}(\theta_{1},\phi_{1},\gamma_{1},\eta_{1}),\ldots ,$ $a_{pol}(\theta_{K},\phi_{K},\gamma_{K},\eta_{K})]\in {\mathbb{C}}^{6\times K}$ is the spatial-polarimetric domain (SPD) steering matrix of a single centralized EMVS. ⊕ is the Khatri-Rao product. $a_{t}(\theta_{k},\phi_{k})$, $a_{r}(\theta_{k},\phi_{k})$ and $a_{pol}(\theta_{k},\phi_{k},\gamma_{k},\eta_{k})$ are expressed as follows:

(2) $$a_{r}(\theta_{k},\phi_{k})=\left[\begin{array}{c} \exp\left(-j\frac{2\pi }{\lambda }\left(x_{r1}\sin\theta_{k}\cos\phi_{k}+y_{r1}\sin\theta_{k}\sin\phi_{k}+z_{r1}\cos\theta_{k}\right)\right)\\ \vdots \\ \exp\left(-j\frac{2\pi }{\lambda }\left(x_{rN-1}\sin\theta_{k}\cos\phi_{k}+y_{rN-1}\sin\theta_{k}\sin\phi_{k}+z_{rN-1}\cos\theta_{k}\right)\right) \end{array}\right]$$

(3) $$a_{t}(\theta_{k},\phi_{k})=\left[\begin{array}{c} \exp\left(-j\frac{2\pi }{\lambda }\left(x_{t1}\sin\theta_{k}\cos\phi_{k}+y_{t1}\sin\theta_{k}\sin\phi_{k}+z_{t1}\cos\theta_{k}\right)\right)\\ \vdots \\ \exp\left(-j\frac{2\pi }{\lambda }\left(x_{tM}\sin\theta_{k}\cos\phi_{k}+y_{tM}\sin\theta_{k}\sin\phi_{k}+z_{tM}\cos\theta_{k}\right)\right) \end{array}\right]$$

(4) $$a_{pol}(\theta_{k},\phi_{k},\gamma_{k},\eta_{k})=\left[\begin{array}{c} e\\ h \end{array}\right]=\left[\begin{array}{c} a_{pol,1}(\theta_{k},\phi_{k},\gamma_{k},\eta_{k})\\ a_{pol,2}(\theta_{k},\phi_{k},\gamma_{k},\eta_{k})\\ a_{pol,3}(\theta_{k},\phi_{k},\gamma_{k},\eta_{k})\\ a_{pol,4}(\theta_{k},\phi_{k},\gamma_{k},\eta_{k})\\ a_{pol,5}(\theta_{k},\phi_{k},\gamma_{k},\eta_{k})\\ a_{pol,6}(\theta_{k},\phi_{k},\gamma_{k},\eta_{k}) \end{array}\right]=\underset{\Theta \left(\theta_{k},\phi_{k}\right)}{\underset{︸}{\left[\begin{array}{cc} \cos\phi_{k}\cos\theta_{k} & -\sin\phi_{k}\\ \sin\phi_{k}\cos\theta_{k} & \cos\phi_{k}\\ -\sin\theta_{k} & 0\\ -\sin\phi_{k} & -\cos\phi_{k}\cos\theta_{k}\\ \cos\phi_{k} & -\sin\phi_{k}\cos\theta_{k}\\ 0 & \sin\theta_{k} \end{array}\right]}}\underset{g\left(\gamma_{k},\eta_{k}\right)}{\underset{︸}{\left[\begin{array}{c} \sin\gamma_{k}e^{j\eta_{k}}\\ \cos\gamma_{k} \end{array}\right]}}$$

where e (the first three elements of $a_{pol}(\theta_{k},\phi_{k},\gamma_{k},\eta_{k})$) and h (the last three elements of $a_{pol}(\theta_{k},\phi_{k},\gamma_{k},\eta_{k})$) represent the electric-field component and magnetic-field component, respectively. They are related to the 2D-DOA and PSA of targets. $b\left(t\right)={\left[\rho_{1}\left(t\right),\ldots ,\rho_{K}\left(t\right)\right]}^{T}\in {\mathbb{C}}^{K\times 1}$ represents reflection coefficient of all targets, where $\rho_{k}\left(t\right)=\alpha_{k}\exp\left(j2\pi f_{k}t\right)$. The amplitude of the reflection coefficient $\alpha_{k}$ is related to the target radar cross section (RCS). The phase of reflection coefficient is related to the Doppler frequency of the target. Note that, here t represents the slow-time. $w\left(t,\tau \right)\in {\mathbb{C}}^{6\left(N-1\right)\times 1}$ represents additive white Gaussian noise whose mean value is zero and covariance matrix is $\sigma_{n}^{2}I_{6\left(N-1\right)}$.

Through matched filtering, the received data can be represented as

(5) $$\begin{array}{cc} X\left(t\right) & =\frac{1}{\Gamma }\sum_{\tau =1}^{\Gamma }x\left(t,\tau \right)s^{H}\left(\tau \right)\\ & =\Lambda_{r}\left[A_{r}\left(\theta ,\phi \right)⊕A_{pol}\left(\theta ,\phi ,\gamma ,\eta \right)\right]\mathrm{diag}\left[b\left(t\right)\right]{\left[\Lambda_{t}A_{t}\left(\theta ,\phi \right)\right]}^{T}\frac{1}{\Gamma }\sum_{\tau =1}^{\Gamma }s\left(\tau \right)s^{H}\left(\tau \right)+N\left(t\right)\\ & =\Lambda_{r}\left[A_{r}\left(\theta ,\phi \right)⊕A_{pol}\left(\theta ,\phi ,\gamma ,\eta \right)\right]\mathrm{diag}\left[b\left(t\right)\right]{\left[\Lambda_{t}A_{t}\left(\theta ,\phi \right)\right]}^{T}+N\left(t\right) \end{array}$$

where $N\left(t\right)=\frac{1}{\Gamma }\sum_{\tau =1}^{\Gamma }w\left(t,\tau \right)s^{H}\left(\tau \right)$. Perform vectorization to (5), we can obtain:

(6) $$y\left(t\right)=\mathrm{vec}\left\{X\left(t\right)\right\}=\Lambda A\left(\theta ,\phi ,\gamma ,\eta \right)b\left(t\right)+n\left(t\right)\in {\mathbb{C}}^{6M\left(N-1\right)\times 1}$$

where $A\left(\theta ,\phi ,\gamma ,\eta \right)=\left[A_{t}\left(\theta ,\phi \right)⊕A_{r}\left(\theta ,\phi \right)⊕A_{pol}\left(\theta ,\phi ,\gamma ,\eta \right)\right]$ $\in {\mathbb{C}}^{6M\left(N-1\right)\times K}$. And its columns are the virtual array steering vector $a\left(\theta ,\phi ,\gamma ,\eta \right)=\left[a_{t}\left(\theta ,\phi \right)⊗a_{r}\left(\theta ,\phi \right)⊗a_{pol}\left(\theta ,\phi ,\gamma ,\eta \right)\right]$. $\Lambda =\Lambda_{t}⊗\Lambda_{r}$. ⊗ is the Kronecker product. And n(t)=vec[N(t)]. vec(·) represents the vectorization operation. The noise corresponding to *m*-th transmitting antenna and the *n*-th receiving antenna is $n_{n,m}=w_{n}s_{m}^{H}$. The elements of the noise covariance matrix are equal to:

(7) $$\begin{array}{cc} E\left[n_{n_{1},m_{1}}\cdot n_{n_{2},m_{2}}^{H}\right] & =E\left\{w_{n_{1}}s_{m_{1}}^{H}\cdot {\left[w_{n_{2}}s_{m_{2}}^{H}\right]}^{H}\right\}\\ & =E\left\{w_{n_{1}}s_{m_{1}}^{H}\cdot s_{m_{2}}w_{n_{2}}^{H}\right\}\\ & =\{\begin{array}{cc} 0, & \mathrm{else}\\ \sigma_{n}^{2}, & n_{1}=n_{2},m_{1}=m_{2} \end{array} \end{array}$$

We can see that n(t) is still a complex white Gaussian noise with zero mean and covariance matrix $\sigma_{n}^{2}I_{6M\left(N-1\right)}$.

When there are no GPU and MC, we can use the method in [26] to estimate the 2D-DOA of targets. For virtual array after matched filtering, it extracts the component with the same orientation in EMVSs to construct a subarray. Then we can obtain six spatially identical subarrays. According to the SPD rotational invariance of the six subarrays, we can get $C_{6}^{2}=15$ rotation invariant factors (RIFs) by using the ESPRIT algorithm. According to the knowledge of [35], we can calculate the incident electromagnetic wave’s electric-field and magnetic-field by only selecting five RIFs. Then we can perform vector cross-product between electric-field and magnetic-field to obtain the pointing vector of source. Last, 2D-DOA can be obtained by the triangle operation with the pointing vector. This is the method which is used in [26] and it is different with the spatial ESPRIT algorithm. It uses the SPD invariance property to construct the electromagnetic component and uses the vector cross-product of these components to get the DOA of targets, while the spatial ESPRIT directly uses the spatial invariance property to estimate the DOA of targets.

When GPU and MC occur, it results in significant distortion of the amplitude and phase of the signals received from the array. This spoils the invariance property of the SPD, so the direct use of the SPD ESPRIT algorithm will lead to wrong estimation of RIFs which will cause DOA estimation errors, therefore, the ESPRIT algorithm in [26] which is computationally efficient cannot be used directly. Based on this, we research the 2D-DOA estimation of MIMO-EMVSs and put forward the ESPRIT-like 2D-DOA estimation algorithm.

## 3. ESPRIT-Like 2D-DOA Estimation Algorithm

The main content of Section 3 includes the following two aspects: firstly, considering the problem of GPU, we propose a novel C-SPD ESPRIT-like algorithm with centralized EMVSs in Section 3.1. It utilizes the ISM to estimate the 2D-DOA and PSA of targets. The C-SPD ESPRIT-like algorithm can obtain good estimation performance without any information of the GPU. Then, taking into account the simultaneous problems of GPU and MC, we revise the C-SPD ESPRIT-algorithm and apply it to a class of separated EMVSs in Section 3.2. This can solve the problem of GPU and MC efficiently.

### 3.1. Centralized Electromagnetic Vector Receiver

**Figure 2:**
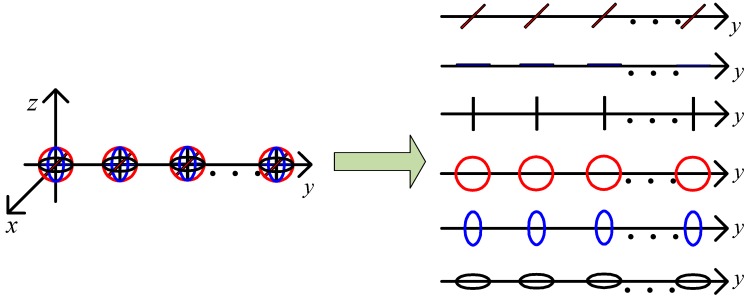
Six spatially identical subarrays offered by EMVSs array.

We propose the C-SPD ESPRIT-like algorithm for when the GPU occurs. It needs a well-calibrated centralized EMVS in the receiving array, so we add a well-calibrated centralized EMVS to the receiving array. To facilitate the derivation, we set it as the first receiver element. If it is not the first element, we can conduct a simple replacement operation on the received data matrix, which makes it become the first array. Then, for virtual array after matched filtering (referencing Equation (5)), we extract the component with the same orient in EMVSs to construct a subarray. We can obtain six spatially identical subarrays. Here we take some EMVSs located in the y axis for example, as shown in Figure 2. Note that the position of EMVS in the receiving array can be arbitrary and it does not affect the later estimation algorithm of DOA.

#### 3.1.1. New Rotational Invariance Property of the SPD with Gain and Phase Error

Here we will derive the new rotational invariance property of the SPD under the condition of GPU.

Define the following vector and matrix:

$$e_{i}^{T}=\left[\underset{i-1}{\underset{︸}{0\;0\;0}}\;1\;\underset{6-i}{\underset{︸}{0\;0}}\right],e_{j}^{T}=\left[\underset{j-1}{\underset{︸}{0\;0\;0}}\;1\;\underset{6-j}{\underset{︸}{0\;0}}\right],\;J_{i}=I_{M}⊗I_{N}⊗e_{i}^{T},\;J_{j}=I_{M}⊗I_{N}⊗e_{j}^{T}.$$

where i and j represent integers between 1 and 6. $e_{i}^{T}$ and $e_{j}^{T}$ are row extraction vector, $J_{i}$ and $J_{j}$ are row extraction matrices which are used to extract the components with the same orientation in EMVSs. These extraction matrices have the following properties:

(8) $$J_{i}J_{i}^{T}=\left[I_{M}⊗I_{N}⊗e_{i}^{T}\right]\cdot {\left[I_{M}⊗I_{N}⊗e_{i}^{T}\right]}^{T}=I_{M}⊗I_{N}⊗1=I_{MN}$$

(9) $$J_{i}^{T}J_{i}={\left[I_{M}⊗I_{N}⊗e_{i}^{T}\right]}^{T}\cdot \left[I_{M}⊗I_{N}⊗e_{i}^{T}\right]=I_{M}⊗I_{N}⊗\mathrm{diag}\left(e_{i}\right)=I_{MN}⊗\mathrm{diag}\left(e_{i}\right)$$

That is to say $J_{i}J_{i}^{T}$ is an MN×MN identity matrix and $J_{i}^{T}J_{i}$ is a diagonal matrix, then utilizing these properties and considering the GPU, we can get the following equation:

(10) $$J_{i}\Lambda A=I_{MN}J_{i}\Lambda A=J_{i}J_{i}^{T}J_{i}\Lambda A=J_{i}\left(J_{i}^{T}J_{i}\right)\Lambda A=J_{i}\Lambda \left(J_{i}^{T}J_{i}\right)A=\left(J_{i}\Lambda J_{i}^{T}\right)J_{i}A$$

The both sides of the equation are multiplied by ${\left(J_{i}\Lambda J_{i}^{T}\right)}^{-1}$, we get:

(11) $$J_{i}A={\left(J_{i}\Lambda J_{i}^{T}\right)}^{-1}J_{i}\Lambda A$$

Similarly, we can get:

(12) $$J_{j}A={\left(J_{j}\Lambda J_{j}^{T}\right)}^{-1}J_{j}\Lambda A$$

When there is no GPU, we know that there is a rotational invariance of SPD (cf. [26]):

(13) $$J_{i}A=J_{j}A\Phi_{i,j}$$

where $\Phi_{i,j}=$ $\mathrm{diag}[a_{pol,i}(\theta_{1},\phi_{1},\gamma_{1},\eta_{1})/a_{pol,j}(\theta_{1},\phi_{1},\gamma_{1},\eta_{1}),\ldots ,$ $a_{pol,i}(\theta_{K},\phi_{K},\gamma_{K},\eta_{K})/a_{pol,j}(\theta_{K},\phi_{K},\gamma_{K},\eta_{K})]$, here we define RIF as $\chi_{k}^{i,j}=a_{pol,i}(\theta_{k},\phi_{k},\gamma_{k},\eta_{k})/a_{pol,j}(\theta_{k},\phi_{k},\gamma_{k},\eta_{k})$.

When there are GPU, substituting Equations (11) and (12) into (13), we get:

(14) $${\left(J_{i}\Lambda J_{i}^{T}\right)}^{-1}J_{i}\Lambda A={\left(J_{j}\Lambda J_{j}^{T}\right)}^{-1}J_{j}\Lambda A\Phi_{i,j}$$

The both sides of the equation are multiplied by $\left(J_{j}\Lambda J_{j}^{T}\right)$, we get the new invariance property of SPD under the condition of gain and phase error:

(15) $$\left(J_{j}\Lambda J_{j}^{T}\right){\left(J_{i}\Lambda J_{i}^{T}\right)}^{-1}J_{i}\Lambda A=J_{j}\Lambda A\Phi_{i,j}$$

The equation looks rather complicated, and we’ll simplify it. Because $J_{i}\Lambda J_{i}^{T}=\left[I_{M}⊗I_{N}⊗e_{i}^{T}\right]\left[\Lambda_{t}⊗\Lambda_{r}\right]\left[I_{M}⊗I_{N}⊗e_{i}\right]=$ $\Lambda_{t}⊗\left\{\left[I_{N}⊗e_{i}^{T}\right]\cdot \Lambda_{r}\cdot \left[I_{N}⊗e_{i}\right]\right\}$ and $J_{j}\Lambda J_{j}^{T}=\Lambda_{t}⊗\left\{\left[I_{N}⊗e_{j}^{T}\right]\cdot \Lambda_{r}\cdot \left[I_{N}⊗e_{j}\right]\right\}$, so the left side of Equation (15) can be simplified as follows:

$$\begin{array}{cc} \left(J_{j}\Lambda J_{j}^{T}\right){\left(J_{i}\Lambda J_{i}^{T}\right)}^{-1} & =\left\{\Lambda_{t}⊗\left[\left(I_{N}⊗e_{j}^{T}\right)\cdot \Lambda_{r}\cdot \left(I_{N}⊗e_{j}\right)\right]\right\}\cdot {\left\{\Lambda_{t}⊗\left[\left(I_{N}⊗e_{i}^{T}\right)\cdot \Lambda_{r}\cdot \left(I_{N}⊗e_{i}\right)\right]\right\}}^{-1}\\ & =\left\{\Lambda_{t}⊗\left[\left(I_{N}⊗e_{j}^{T}\right)\cdot \Lambda_{r}\cdot \left(I_{N}⊗e_{j}\right)\right]\right\}\cdot \left\{\Lambda_{t}^{-1}⊗{\left[\left(I_{N}⊗e_{i}^{T}\right)\cdot \Lambda_{r}\cdot \left(I_{N}⊗e_{i}\right)\right]}^{-1}\right\}\\ & =I_{M}⊗\left\{\left[\left(I_{N}⊗e_{j}^{T}\right)\cdot \Lambda_{r}\cdot \left(I_{N}⊗e_{j}\right)\right]\cdot {\left[\left(I_{N}⊗e_{i}^{T}\right)\cdot \Lambda_{r}\cdot \left(I_{N}⊗e_{i}\right)\right]}^{-1}\right\}\\ & =\mathrm{diag}\left(c_{ri,j}\right) \end{array}$$

where $c_{ri,j}={\vec{1}}_{M}⊗\left[d_{rj}⊙\frac{1}{d_{ri}}\right]={\vec{1}}_{M}⊗\left[d_{rj}⊙d_{ri}⊙\frac{1}{d_{ri}^{2}}\right]$, ${\vec{1}}_{M}={\left[\underset{M}{\underset{︸}{1,1,\ldots ,1}}\right]}^{T}$. $d_{ri}=\mathrm{diag}\left[\left(I_{N}⊗e_{i}^{T}\right)\Lambda_{r}\left(I_{N}⊗e_{i}\right)\right]$ is a vector which contains the gain and phase error of the *i*-th subarray. Substituting it into Equation (15), we get:

(16) $$\mathrm{diag}\left(c_{ri,j}\right)J_{i}\Lambda A=J_{j}\Lambda A\Phi_{i,j}$$

This is the new invariance property of SPD under the condition of gain and phase error.

#### 3.1.2. Closed form Solution of RIFs

Here, we will derive the closed form solution of RIFs based on Equation (16). The covariance matrix of received data can be estimated by:

(17) $$\hat{R}=1/L\sum_{i=1}^{L}y\left(t\right)\cdot y^{H}\left(t\right)$$

By eigen-decomposition, we can obtain:

(18) $$\hat{R}=U_{s}\Sigma_{s}U_{s}^{H}+\sigma_{n}^{2}U_{n}U_{n}^{H}$$

(19) $$U_{s}=\Lambda AT$$

where $U_{s}=\Lambda AT$ is the signal subspace

Referring Equations (11) and (12), define $U_{ri}$ and $U_{rj}$ as the *i*-th and *j*-th components of the signal subspace which can be represented as follows:

(20) $$U_{ri}=J_{i}U_{s}$$

(21) $$U_{rj}=J_{j}U_{s}$$

Substituting Equation (19) into Equation (16), we can get the new invariance property of signal space under the condition of gain and phase error:

(22) $$\mathrm{diag}\left(c_{ri,j}\right)U_{ri}=U_{rj}T_{i,j}{}^{-1}\Phi_{i,j}T_{i,j}=U_{rj}\Psi_{i,j}$$

where $\Psi_{i,j}=T_{i,j}{}^{-1}\Phi_{i,j}T_{i,j}$.

Because $\mathrm{diag}\left(c_{ri,j}\right)$ and $\Psi_{i,j}$ are unknown, the solution of Equation (22) can be got by solving the following constrained optimization problem:

(23) $$\left\{{\hat{c}}_{ri,j},{\hat{\Psi }}_{i,j}\right\}=\underset{c_{ri,j},\Psi_{i,j}}{\mathrm{argmin}}{\;\|\mathrm{diag}\left(c_{ri,j}\right)U_{ri}-U_{rj}\Psi_{i,j}\|}_{F}^{2}\;s.t.\varepsilon_{1}^{T}c_{ri,j}=1\;$$

where $\varepsilon_{1}={\left[1,\overset{1\times \left(MN-1\right)}{\overset{︷}{0,\ldots ,0}}\right]}^{T}$. According to the least squares method, the solution of Equation (23) is:

(24) $${\hat{\Psi }}_{i,j}={\left(U_{rj}^{H}U_{rj}\right)}^{-1}U_{rj}^{H}\mathrm{diag}\left(c_{ri,j}\right)U_{ri}$$

By substituting Equation (24) into (23), the optimization problem can be translated into:

(25) $${\hat{c}}_{ri,j}=\underset{c_{ri,j}}{\mathrm{argmin}}{\;\|P_{U_{rj}}^{⊥}\mathrm{diag}\left(c_{ri,j}\right)U_{ri}\|}_{F}^{2}\;s.t.\varepsilon_{1}^{T}c_{ri,j}=1\;$$

where $P_{U_{rj}}^{⊥}=I_{MN}-U_{rj}{\left(U_{rj}^{H}U_{rj}\right)}^{-1}U_{rj}^{H}$.

According to the related knowledge of matrix theory, we can simplify the objective function as follows:

(26) $$\begin{array}{cc} {\left\|P_{U_{rj}}^{⊥}\mathrm{diag}\left(c_{ri,j}\right)U_{ri}\right\|}_{F}^{2} & =\mathrm{tr}\left\{U_{ri}^{H}\mathrm{diag}\left(c_{ri,j}^{H}\right)P_{U_{rj}}^{⊥}\mathrm{diag}\left(c_{ri,j}\right)U_{ri}\right\}\\ & =\mathrm{tr}\left\{P_{U_{rj}}^{⊥}\mathrm{diag}\left(c_{ri,j}\right)U_{ri}U_{ri}^{H}\mathrm{diag}\left(c_{ri,j}^{H}\right)\right\}\\ & =c_{ri,j}^{H}\left[P_{U_{rj}}^{⊥}⊙{\left(U_{ri}U_{ri}^{H}\right)}^{T}\right]c_{ri,j}\\ & =c_{ri,j}^{H}Q_{ri,j}c_{ri,j} \end{array}$$

where $Q_{ri,j}=P_{U_{rj}}^{⊥}⊙{\left(U_{ri}U_{ri}^{H}\right)}^{T}$. ⊙ denotes the Hadamard product. Now we get the new optimazition problem:

(27) $${\hat{c}}_{ri,j}=\underset{c_{ri,j}}{\mathrm{argmin}\;}c_{ri,j}^{H}Q_{ri,j}c_{ri,j}\;s.t.\varepsilon_{1}^{T}c_{ri,j}=1\;$$

According to the Lagrange multipliers method, the solution of (27) is:

(28) $${\hat{c}}_{ri,j}=\frac{Q_{ri,j}^{-1}\varepsilon_{1}}{\varepsilon_{1}^{T}Q_{ri,j}^{-1}\varepsilon_{1}}\;$$

Substituting Equation (28) into (24), we can get the estimation as follow:

(29) $${\hat{\Psi }}_{i,j}={\left(U_{rj}^{H}U_{rj}\right)}^{-1}U_{rj}^{H}\cdot \mathrm{diag}\left(\frac{Q_{ri,j}^{-1}\varepsilon_{1}}{\varepsilon_{1}^{T}Q_{ri,j}^{-1}\varepsilon_{1}}\right)\cdot U_{ri}$$

Then, performing eigen-decompostion to it, estimation of RIFs can be obtained. Note that $Q_{ri,j}$ is required to be nonsingular to estimate the vector ${\hat{c}}_{ri,j}$. In the case of infinite snapshots, $Q_{ri,j}$ is a nonsingular matrix. To ensure that $Q_{ri,j}$ is also a nonsingular matrix with finite snapshots in practice, diagonal loading is a possible method to handle this problem. In addition, from the extensive experiments with finite snapshots that we have made, the matrix $Q_{ri,j}$ is always nonsingular. Thus, it is not necessary to use the diagonal loading method for $Q_{ri,j}$ in general.

#### 3.1.3. Estimation of 2D-DOA and PSA

Because RIFs $\chi_{k}^{i,j}|i,j=1,\ldots ,6$ in different $\Phi_{i,j}$ have different orders. So we should pair these RIFs. Note that, RIFs are matched with their eigenvectors. Thus, we can pair these RIFs for the identical target by pairing the orthogonal rows of $T_{i,j}$. And the procedure is omitted here and interested readers can refer to the [26].

Because i,j=1,…,6, so here are $C_{6}^{2}=15$ choices for RIFs $\chi_{k}^{i,j}$. Set i=j+1. That is to say, we pick five from them.

And the relationship between these polarization components can be represented as follows:

(30) $$a_{pol,2}\left(\theta_{k},\phi_{k},\gamma_{k},\eta_{k}\right)=a_{pol,1}\left(\theta_{k},\phi_{k},\gamma_{k},\eta_{k}\right)\cdot \chi_{k}^{2,1}\;a_{pol,3}\left(\theta_{k},\phi_{k},\gamma_{k},\eta_{k}\right)=a_{pol,1}\left(\theta_{k},\phi_{k},\gamma_{k},\eta_{k}\right)\cdot \chi_{k}^{3,1}\;a_{pol,4}\left(\theta_{k},\phi_{k},\gamma_{k},\eta_{k}\right)=a_{pol,1}\left(\theta_{k},\phi_{k},\gamma_{k},\eta_{k}\right)\cdot \chi_{k}^{4,1}\;a_{pol,5}\left(\theta_{k},\phi_{k},\gamma_{k},\eta_{k}\right)=a_{pol,1}\left(\theta_{k},\phi_{k},\gamma_{k},\eta_{k}\right)\cdot \chi_{k}^{5,1}\;a_{pol,6}\left(\theta_{k},\phi_{k},\gamma_{k},\eta_{k}\right)=a_{pol,1}\left(\theta_{k},\phi_{k},\gamma_{k},\eta_{k}\right)\cdot \chi_{k}^{6,1}$$

Then, the Pointing vector can be obtained by the cross-multiplication between the electric-field component and the magnetic-field component based on Maxwell equation:

(31) $$\Gamma =e\times h=\left[\begin{array}{c} a_{pol,1}\left(\theta_{k},\phi_{k},\gamma_{k},\eta_{k}\right)\\ a_{pol,2}\left(\theta_{k},\phi_{k},\gamma_{k},\eta_{k}\right)\\ a_{pol,3}\left(\theta_{k},\phi_{k},\gamma_{k},\eta_{k}\right) \end{array}\right]\times \left[\begin{array}{c} a_{pol,4}\left(\theta_{k},\phi_{k},\gamma_{k},\eta_{k}\right)\\ a_{pol,5}\left(\theta_{k},\phi_{k},\gamma_{k},\eta_{k}\right)\\ a_{pol,6}\left(\theta_{k},\phi_{k},\gamma_{k},\eta_{k}\right) \end{array}\right]$$

We know that there is a relationship between Pointing vector and the 2D-DOA of target:

(32) $$\Gamma =\left[\begin{array}{c} u\\ v\\ w \end{array}\right]=\left[\begin{array}{c} \sin\theta \cos\phi \\ \sin\theta \sin\phi \\ \cos\theta \end{array}\right]$$

Combing Equations (30)–(32), we have

(33) $${\left\|a_{pol,1}\left(\theta_{k},\phi_{k},\gamma_{k},\eta_{k}\right)\right\|}^{2}\left[\begin{array}{c} 1\\ \chi_{k}^{2,1}\\ \chi_{k}^{3,1} \end{array}\right]\times \left[\begin{array}{c} \chi_{k}^{4,1}\\ \chi_{k}^{5,1}\\ \chi_{k}^{6,1} \end{array}\right]=\left[\begin{array}{c} u\\ v\\ w \end{array}\right]$$

Then we can get the estimation of direction cosines $\hat{u}$ $\hat{v}$ $\hat{w}$ by normalized processing to the result of Equation (33). Last, we can get the 2D-DOA estimation through the following triangulation:

(34) $$\left\{ \begin{array}{ll} {\hat{\theta }}_{k}=\arcsin\left(\sqrt{{\hat{u}}_{k}^{2}+{\hat{v}}_{k}^{2}}\right), & if\;{\hat{v}}_{k}\geq 0\\ {\hat{\theta }}_{k}=-\arcsin\left(\sqrt{{\hat{u}}_{k}^{2}+{\hat{v}}_{k}^{2}}\right)+\pi , & if\;{\hat{v}}_{k}<0\\ {\hat{\phi }}_{k}=\arctan\left(\frac{{\hat{v}}_{k}}{{\hat{u}}_{k}}\right), & \mathrm{if}\;{\hat{u}}_{k}\geq 0\\ {\hat{\phi }}_{k}=\arctan\left(\frac{{\hat{v}}_{k}}{{\hat{u}}_{k}}\right)+\pi , & \mathrm{if}\;{\hat{u}}_{k}<0 \end{array}\right.,k=1,\ldots ,K$$

None of the above processing uses the antenna location information. So it is suitable for arbitrary array configuration.

Then, according to Equation (4), we get

(35) $$g\left(\gamma_{k},\eta_{k}\right)={\left[\Theta^{H}\left(\theta_{k},\phi_{k}\right)\Theta \left(\theta_{k},\phi_{k}\right)\right]}^{-1}\Theta \left(\theta_{k},\phi_{k}\right)\left[a_{pol}(\theta_{k},\phi_{k},\gamma_{k},\eta_{k})\right]$$

Substitute $\left({\hat{\theta }}_{k}\;{\hat{\phi }}_{k}\right)$ into the above equation, the corresponding PSA can be got by:

(36) $$\left\{ \begin{array}{l} {\hat{\gamma }}_{k}=\arctan\left|\frac{{\left[\hat{g}\left(\gamma_{k},\eta_{k}\right)\right]}_{1}}{{\left[\hat{g}\left(\gamma_{k},\eta_{k}\right)\right]}_{2}}\right|\\ {\hat{\eta }}_{k}=∠{\left[\hat{g}\left(\gamma_{k},\eta_{k}\right)\right]}_{1}-∠{\left[\hat{g}\left(\gamma_{k},\eta_{k}\right)\right]}_{2} \end{array}\right.$$

According to the above analysis, the estimation of 2D-DOA and PSA for monostatic MIMO radar with centralized EMVSs under GPU can be summarized as follows:

Step 1. Perform matched filtering and vectorization to the received data by (5) and (6);
Step 2. Calculate the covariance matrix of virtual array by (17). And perform the eigen-decomposition to it to get the signal subspace by (18);
Step 3. Compute the estimation of the relative GPU of the transmit and receive sensors by (28). Substituting the result into (24), get the estimation of $\Psi_{i,j}$, then compute the eigenvalues of $\Psi_{i,j}$ to obtain the RIFs estimations $\chi_{k}^{i,j}|i,j=1,\ldots ,6$;
Step 4. Pairing the estimation of RIFs $\left\{\chi_{k}^{2,1},\chi_{k}^{3,2}\chi_{k}^{4,3}\chi_{k}^{5,4}\chi_{k}^{6,5},\left(k=1,\ldots ,K\right)\right\}$ for the same target by the connection between eigenvalues and corresponding eigenvectors;
Step 5. Implementing the vector cross product by (33) based on to the paired estimations RIFs $\left\{\chi_{k}^{2,1},\chi_{k}^{3,2}\chi_{k}^{4,3}\chi_{k}^{5,4}\chi_{k}^{6,5},\left(k=1,\ldots ,K\right)\right\}$. And get the estimation of direction cosines by normalization processing;
Step 6. Last, we can get the 2D-DOA estimations by (34). And the auxiliary polarization angle and polarization phase difference can be obtained by (36).

From the above analysis, we can see that the algorithm has the following advantages:

1. Anti gain and phase error unknown;
2. Suitable for any configuration;
3. Similarly to the ESPRIT method, the calculation is small without angle searching;
4. The angle of the whole airspace can be estimated;
5. It is applicable to multiple EMVSs at the receiver;
6. *MN* targets can be estimated at most.

### 3.2. Separated Electromagnetic Vector Receiver

**Figure 3:**
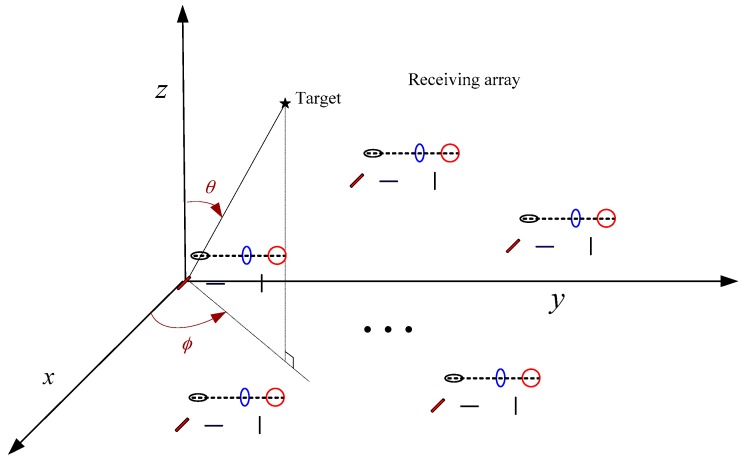
Monostatic MIMO radar with arbitrarily spaced separated EMVSs.

In the above analysis, we did not consider the MC between components of the centralized EMVS. When the MC and GPU coexist between the components of the centralized EMVS, the centralized EMVSs cannot get good estimation results due to the serious problem of mutual coupling. One way to solve this problem is to adopt separated EMVSs which are shown in Figure 3. The separated EMVSs can reduce the influence of MC, but the algorithm we proposed in Section 3.1 may not be suitable, and we need to do some revision. Besides, in order to make our algorithm feasible, the structure of separated EMVS may need to meet certain conditions. Now we will revise the algorithm and derive a class of separated EMVSs which are satisfied with the requirements. The spatial phase shift factors of separated electromagnetic vector sensor with arbitrary structure are listed in Table 1.

The most difference between the separated EMVS and the centralized EMVS is that the polarization steer matrix changed. The polarization steer matrix of separated EMVS is:

(37) $${\tilde{a}}_{pol}(\theta_{k},\phi_{k},\gamma_{k},\eta_{k})=\left[\begin{array}{c} \tilde{e}\\ \tilde{h} \end{array}\right]=\left[\begin{array}{c} {\tilde{e}}_{x}\\ {\tilde{e}}_{y}\\ {\tilde{e}}_{z}\\ {\tilde{h}}_{x}\\ {\tilde{h}}_{y}\\ {\tilde{h}}_{z} \end{array}\right]=\left[\begin{array}{c} d_{ex}\cdot a_{pol,1}(\theta_{k},\phi_{k},\gamma_{k},\eta_{k})\\ d_{ey}\cdot a_{pol,2}(\theta_{k},\phi_{k},\gamma_{k},\eta_{k})\\ d_{ez}\cdot a_{pol,3}(\theta_{k},\phi_{k},\gamma_{k},\eta_{k})\\ d_{hx}\cdot a_{pol,4}(\theta_{k},\phi_{k},\gamma_{k},\eta_{k})\\ d_{hy}\cdot a_{pol,5}(\theta_{k},\phi_{k},\gamma_{k},\eta_{k})\\ d_{hz}\cdot a_{pol,6}(\theta_{k},\phi_{k},\gamma_{k},\eta_{k}) \end{array}\right]=d(u_{k},v_{k})⊙a_{pol}(\theta_{k},\phi_{k},\gamma_{k},\eta_{k})$$

As we can see, each component of EMVS has added a phase shift factor. Then, under the gain and phase error condition, the rotation invariance of SPD is transformed into the following form:

$$\mathrm{diag}\left(c_{ri,j}\right)J_{i}\Lambda A=J_{j}\Lambda A{\bar{\Phi }}_{i,j}$$

Compared with Equation (16), the RIFs have changed. The original RIFs is $\Phi_{i,j}=$ $\mathrm{diag}[a_{pol,i}(\theta_{1},\phi_{1},\gamma_{1},\eta_{1})/a_{pol,j}(\theta_{1},\phi_{1},\gamma_{1},\eta_{1}),\ldots ,$ $a_{pol,i}(\theta_{K},\phi_{K},\gamma_{K},\eta_{K})/a_{pol,j}(\theta_{K},\phi_{K},\gamma_{K},\eta_{K})]$. The new RIFs become $\Phi_{i,j}=\mathrm{diag}$$\left\{\begin{array}{l} \left[d_{i,1}\cdot a_{pol,i}(\theta_{1},\phi_{1},\gamma_{1},\eta_{1})\right]/\left[d_{j,1}\cdot a_{pol,j}(\theta_{1},\phi_{1},\gamma_{1},\eta_{1})\right]\\ ,\ldots ,\left[d_{i,K}\cdot a_{pol,i}(\theta_{K},\phi_{K},\gamma_{K},\eta_{K})\right]/\left[d_{j,K}\cdot a_{pol,j}(\theta_{K},\phi_{K},\gamma_{K},\eta_{K})\right] \end{array}\right\}$, but this doesn’t affect the solution of new RIFs. That is to say, the algorithm we proposed in Section 3.1 still can be used to estimate the new RIFs, so if there is a well-calibrated separated EMVS in the receiving array, the new RIFs still can be estimated by the eigen-decompostion of Equation (29).

The 2D-DOA estimation relies on the vector cross product of electric-field components and magnetic-field components which are reconstructed based on RIFs. Because the RIFs have changed, so the vector cross product of separated EMVSs are changed to the new form which is shown in Equation (38).

The vector cross product result shows that each component contains two “phase shift factors”. This makes it very difficult to estimate the DOA of targets. We discovered that if the two phase shift factors are equal, i.e., Equations (39)–(41) are shown, the polarization parameter in the results of vector cross product can be eliminated. This is very favorable to our estimation of targets’ DOA. The reason of eliminating the polarization parameters is to reduce the unknown quantity contained in the equation, and to facilitate the estimation of azimuth and elevation angle. The condition is not a necessary condition, that is, array design does not meet the needs of the elimination of polarization parameters.

(38) $$\begin{array}{l} \tilde{\Gamma }=\tilde{e}\times {\tilde{h}}^{*}=\left[\begin{array}{c} {\tilde{e}}_{x}\\ {\tilde{e}}_{y}\\ {\tilde{e}}_{z} \end{array}\right]\times \left[\begin{array}{c} {\tilde{h}}_{x}^{*}\\ {\tilde{h}}_{y}^{*}\\ {\tilde{h}}_{z}^{*} \end{array}\right]=\left[\begin{array}{ccc} 0 & -{\tilde{e}}_{z} & {\tilde{e}}_{y}\\ {\tilde{e}}_{z} & 0 & -{\tilde{e}}_{x}\\ -{\tilde{e}}_{y} & {\tilde{e}}_{x} & 0 \end{array}\right]\left[\begin{array}{c} {\tilde{h}}_{x}^{*}\\ {\tilde{h}}_{y}^{*}\\ {\tilde{h}}_{z}^{*} \end{array}\right]\\ =\left[\begin{array}{l} \left(\sin\theta \cos\phi \sin^{2}\gamma -\sin\theta \cos\theta \sin\phi \sin\gamma \cos\gamma e^{j\eta }\right)\\ \cdot e^{j\frac{2\pi }{\lambda }\left[\left(x_{hy}-x_{ez}\right)u+\left(y_{hy}-y_{ez}\right)v+\left(z_{hy}-z_{ez}\right)w\right]}\\ +\left(\sin\theta \cos\phi \cos^{2}\gamma +\sin\theta \cos\theta \sin\phi \sin\gamma \cos\gamma e^{j\eta }\right)\\ \cdot e^{j\frac{2\pi }{\lambda }\left[\left(x_{hz}-x_{ey}\right)u+\left(y_{hz}-y_{ey}\right)v+\left(z_{hz}-z_{ey}\right)w\right]}\\ \left(\sin\theta \sin\phi \sin^{2}\gamma +\sin\theta \cos\theta \cos\phi \sin\gamma \cos\gamma e^{j\eta }\right)\\ \cdot e^{j\frac{2\pi }{\lambda }\left[\left(x_{hx}-x_{ez}\right)u+\left(y_{hx}-y_{ez}\right)v+\left(z_{hx}-z_{ez}\right)w\right]}\\ +\left(\sin\theta \sin\phi \cos^{2}\gamma -\sin\theta \cos\theta \sin\phi \sin\gamma \cos\gamma e^{j\eta }\right)\\ \cdot e^{j\frac{2\pi }{\lambda }\left[\left(x_{hz}-x_{ex}\right)u+\left(y_{hz}-y_{ex}\right)v+\left(z_{hz}-z_{ex}\right)w\right]}\\ \left[\cos\theta \left(\sin^{2}\phi \sin^{2}\gamma +\cos^{2}\phi \cos^{2}\gamma \right)+\sin\phi \cos\phi \sin\gamma \cos\gamma \left(e^{-j\eta }+\cos^{2}\theta e^{j\eta }\right)\right]\\ \cdot e^{j\frac{2\pi }{\lambda }\left[\left(x_{hx}-x_{ey}\right)u+\left(y_{hx}-y_{ey}\right)v+\left(z_{hx}-z_{ey}\right)w\right]}\\ +\left[\cos\theta \left(\cos^{2}\phi \sin^{2}\gamma +\sin^{2}\phi \cos^{2}\gamma \right)-\sin\phi \cos\phi \sin\gamma \cos\gamma \left(e^{-j\eta }+\cos^{2}\theta e^{j\eta }\right)\right]\\ \cdot e^{j\frac{2\pi }{\lambda }\left[\left(x_{hy}-x_{ex}\right)u+\left(y_{hy}-y_{ex}\right)v+\left(z_{hy}-z_{ex}\right)w\right]} \end{array}\right] \end{array}$$

(39) $$e^{j\frac{2\pi }{\lambda }\left[\left(x_{hy}-x_{ez}\right)u+\left(y_{hy}-y_{ez}\right)v+\left(z_{hy}-z_{ez}\right)w\right]}=e^{j\frac{2\pi }{\lambda }\left[\left(x_{hz}-x_{ey}\right)u+\left(y_{hz}-y_{ey}\right)v+\left(z_{hz}-z_{ey}\right)w\right]}$$

(40) $$e^{j\frac{2\pi }{\lambda }\left[\left(x_{hx}-x_{ez}\right)u+\left(y_{hx}-y_{ez}\right)v+\left(z_{hx}-z_{ez}\right)w\right]}=e^{j\frac{2\pi }{\lambda }\left[\left(x_{hz}-x_{ex}\right)u+\left(y_{hz}-y_{ex}\right)v+\left(z_{hz}-z_{ex}\right)w\right]}$$

(41) $$e^{j\frac{2\pi }{\lambda }\left[\left(x_{hx}-x_{ey}\right)u+\left(y_{hx}-y_{ey}\right)v+\left(z_{hx}-z_{ey}\right)w\right]}=e^{j\frac{2\pi }{\lambda }\left[\left(x_{hy}-x_{ex}\right)u+\left(y_{hy}-y_{ex}\right)v+\left(z_{hy}-z_{ex}\right)w\right]}$$

According to Equations (39)–(41), we can derive the following structural relationship of components of EMVS respectively:

(42) $$\{\begin{array}{c} \left(x_{hy}-x_{ez}\right)=\left(x_{hz}-x_{ey}\right)\\ \left(y_{hy}-y_{ez}\right)=\left(y_{hz}-y_{ey}\right)\\ \left(z_{hy}-z_{ez}\right)=\left(z_{hz}-z_{ey}\right) \end{array}$$

(43) $$\{\begin{array}{c} \left(x_{hx}-x_{ez}\right)=\left(x_{hz}-x_{ex}\right)\\ \left(y_{hx}-y_{ez}\right)=\left(y_{hz}-y_{ex}\right)\\ \left(z_{hx}-z_{ez}\right)=\left(z_{hz}-z_{ex}\right) \end{array}$$

(44) $$\{\begin{array}{c} \left(x_{hx}-x_{ey}\right)=\left(x_{hy}-x_{ex}\right)\\ \left(y_{hx}-y_{ey}\right)=\left(y_{hy}-y_{ex}\right)\\ \left(z_{hx}-z_{ey}\right)=\left(z_{hy}-z_{ex}\right) \end{array}$$

Equation (44) can be derived by Equations (42) and (43), so the final form of the structure of EMVS can be represented as:

(45) $$\{\begin{array}{c} \vec{HyEz}=\vec{HzEy}\\ \vec{HxEz}=\vec{HzEx} \end{array}$$

Equation (45) is a simplified form of Equations (42) and (43). Equation (38) acquires the following form:

(46) $$\tilde{\Gamma }=\left[\begin{array}{l} ue^{j\frac{2\pi }{\lambda }\left[\left(x_{hy}-x_{ez}\right)u+\left(y_{hy}-y_{ez}\right)v+\left(z_{hy}-z_{ez}\right)w\right]}\\ ve^{j\frac{2\pi }{\lambda }\left[\left(x_{hx}-x_{ez}\right)u+\left(y_{hx}-y_{ez}\right)v+\left(z_{hx}-z_{ez}\right)w\right]}\\ we^{j\frac{2\pi }{\lambda }\left[\left(x_{hx}-x_{ey}\right)u+\left(y_{hx}-y_{ey}\right)v+\left(z_{hx}-z_{ey}\right)w\right]} \end{array}\right]=\left[\begin{array}{l} ue^{j\frac{2\pi }{\lambda }\left[\left(x_{hz}-x_{ey}\right)u+\left(y_{hz}-y_{ey}\right)v+\left(z_{hz}-z_{ey}\right)w\right]}\\ ve^{j\frac{2\pi }{\lambda }\left[\left(x_{hz}-x_{ex}\right)u+\left(y_{hz}-y_{ex}\right)v+\left(z_{hz}-z_{ex}\right)w\right]}\\ we^{j\frac{2\pi }{\lambda }\left[\left(x_{hy}-x_{ex}\right)u+\left(y_{hy}-y_{ex}\right)v+\left(z_{hy}-z_{ex}\right)w\right]} \end{array}\right]$$

It shows that the absolute value of the direction cosine estimation in all directions can be obtained by the modulus of the Equation (46). Positive and negative determination of directional cosine estimation may require some prior information. Unless additional conditions are added, we can estimate hemispheric airspace at most. If we get the true value of these direction cosine based on prior information, the estimation result is as follows:

(47) $$\left\{ \begin{array}{ll} {\hat{\theta }}_{k}=\arcsin\left(\sqrt{{\hat{u}}_{k}^{2}+{\hat{v}}_{k}^{2}}\right), & if\;{\hat{v}}_{k}\geq 0\\ {\hat{\theta }}_{k}=-\arcsin\left(\sqrt{{\hat{u}}_{k}^{2}+{\hat{v}}_{k}^{2}}\right)+\pi , & if\;{\hat{v}}_{k}<0\\ {\hat{\phi }}_{k}=\arctan\left(\frac{{\hat{v}}_{k}}{{\hat{u}}_{k}}\right), & \mathrm{if}\;{\hat{u}}_{k}\geq 0\\ {\hat{\phi }}_{k}=\arctan\left(\frac{{\hat{v}}_{k}}{{\hat{u}}_{k}}\right)+\pi , & \mathrm{if}\;{\hat{u}}_{k}<0 \end{array}\right.,k=1,\ldots ,K$$

According to Equation (37), we can get the following equation:

(48) $$g\left(\gamma_{k},\eta_{k}\right)={\left[\Theta^{H}\left(\theta_{k},\phi_{k}\right)\Theta \left(\theta_{k},\phi_{k}\right)\right]}^{-1}\Theta \left(\theta_{k},\phi_{k}\right)\left[{\tilde{a}}_{pol}(\theta_{k},\phi_{k},\gamma_{k},\eta_{k})⊙d^{*}\left(u_{k},v_{k}\right)\right]$$

Note that, compared with Equation (35), there is a phase shift factor in the Equation (48). Substitute $({\hat{\theta }}_{k},$ ${\hat{\phi }}_{k})$ into the above equation, we can get the estimation of PSA:

(49) $$\left\{ \begin{array}{l} {\hat{\gamma }}_{k}=\arctan\left|\frac{{\left[\hat{g}\left(\gamma_{k},\eta_{k}\right)\right]}_{1}}{{\left[\hat{g}\left(\gamma_{k},\eta_{k}\right)\right]}_{2}}\right|\\ {\hat{\eta }}_{k}=∠\hat{g}{\left(\gamma_{k},\eta_{k}\right)}_{1}-∠\hat{g}{\left(\gamma_{k},\eta_{k}\right)}_{2} \end{array}\right.$$

Note that, the structure of the EMVS needs to satisfy Equation (45), but the array configuration still can be arbitrary.

To differentiate from the C-SPD ESPRIT-like algorithm of centralized EMVSs which we proposed in Section 3.1, we call this method as spatial-polarimetric domain ESPRIT-like algorithm based on separated EMVS (S-SPD ESPRIT-like algorithm). Where ‘C’ and ‘S’ represent ‘centralized’ and ‘separated’, respectively.

## 4. Comparison of Advantages and Disadvantages of Each Method

Here we compare our algorithm with several other error correction algorithms. The method in [32,33] can only calibrate one error, so when the two errors occur simultaneously, it will seriously affect the performance of the two methods. Besides, the method in [32] is a search-based algorithm which has large computational complexity. The method in [34] can tackle GPU and MC at the same time, but it only suitable for ULA or UCA. Our proposed method can solve the GPU and MC problem of MIMO-EMVS simultaneously and the array configuration can be arbitrary. That is to say, when the array is not ULA or UCA, the method in [34] will be invalid and our method is still able to work. Therefore, our method has wider applicability. Our method is based on the ESPRIT algorithm, so our method also has low complexity. Because we use the rotational invariance of spatial-polarimetric domain and don’t use the array aperture, so there will a certain distance between the RMSE curve of our method and the Cramér-Rao Bound (CRB). The comparison of algorithm of Ref. [26], C-SPD ESPRIT-like algorithm and S-SPD ESPRIT-like algorithm is listed in Table 2.

Note that none of the above methods requires any angle searching process. These algorithms are based on the ESPRIT algorithm, so they all have low complexity. Note that, both the C-SPD ESPRIT-like algorithm and S-SPD ESPRIT-like algorithm are suitable for arbitrary array configuration. That is because we use the connection between these electromagnetic components to estimate DOA which has no requirement for array configuration.

## 5. Numerical Results

In this section, we will perform several simulation experiments to test the effectiveness of our algorithms. Assume there is a MIMO radar with M=4 and N=4. The transmitting and receiving sensors are set on the *x*-axis and *y*-axis, respectively. The spacing between adjacent element is half the wavelength. Therefore the MIMO radar consists of an L-shape array. Note that the MIMO radar can take an arbitrary configuration.

**Figure 4:**
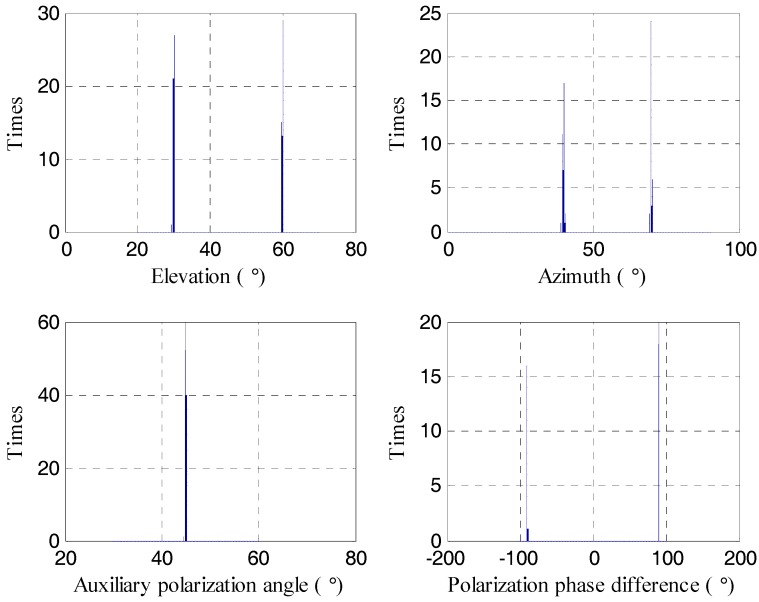
The estimation result of the proposed C-SPD ESPRIT-like algorithm.

**Figure 5:**
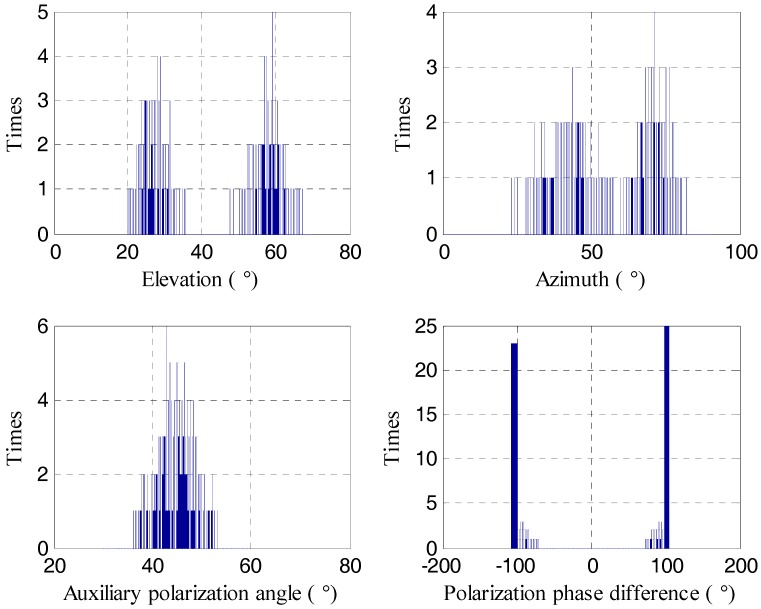
The estimation result of the algorithm of [26].

In the first simulation, we show the estimation performance of the proposed C-SPD ESPRIT-like algorithm. Assume there are two targets which incident from the angle $\left(\theta_{1},\phi_{1}\right)=\left(30{}^{\circ},40{}^{\circ}\right)$ and $\left(\theta_{2},\phi_{2}\right)=\left(60{}^{\circ},70{}^{\circ}\right)$. Their PSA values are $\left(\gamma_{1},\eta_{1}\right)=\left(45{}^{\circ},90{}^{\circ}\right)$ and $\left(\gamma_{2},\eta_{2}\right)=\left(45{}^{\circ},-90{}^{\circ}\right)$. That is to say the first target is left-circularly polarized and the second target is right-circularly polarized. In this simulation, assume that SNR = 20 dB and the number of snapshots is 1000. Figure 4 and Figure 5 show the histograms of estimation results by our algorithm and algorithm of [26], respectively. Five hundred Monte Carlo trials are performed with each algorithm. We can see that the proposed algorithm can accurately estimate the parameters of the target which verifies the correctness of the proposed 2D-DOA estimation algorithm. Meanwhile, the performance of method in [26] is poor.

**Figure 6:**
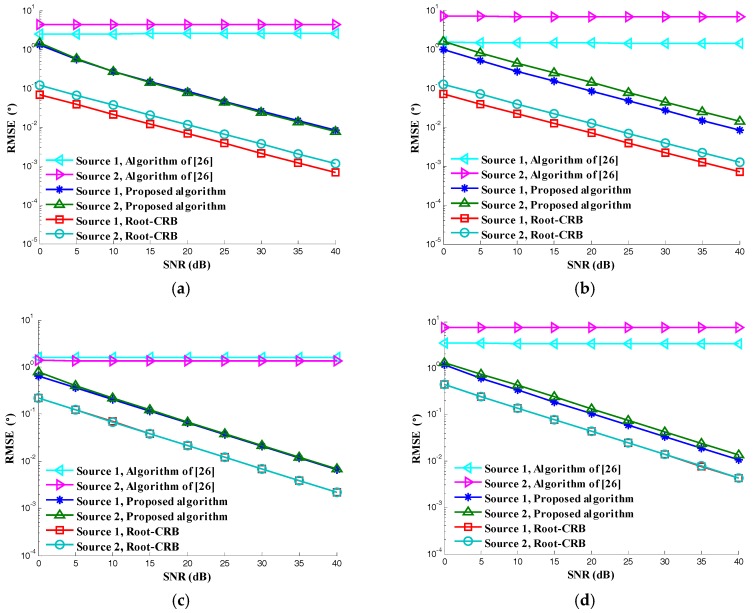
RMSE versus SNR: (**a**) Elevation; (**b**) Azimuth; (**c**) Auxiliary polarization angle; (**d**) Polarization phase difference.

In the second simulation, we compare the estimation accuracy of the proposed C-SPD ESPRIT-like algorithm. The parameters are same as in the first simulation. The SNR varies from 0 dB to 40 dB. Two hundred simulations are conducted under each SNR. The simulation result is shown in Figure 6. As we can see, the C-SPD ESPRIT-like algorithm is getting better and better with the increase of SNR. There is a gap between the proposed C-SPD ESPRIT-like algorithm and CRB (for a detailed derivation, see Appendix A), while the performance of the method in [26] is poor no matter how much the SNR increases. The reason for the existence of the gap is that the C-SPD ESPRIT-like algorithm only utilizes the information inside the vector sensor and the array aperture is not utilized.

**Figure 7:**
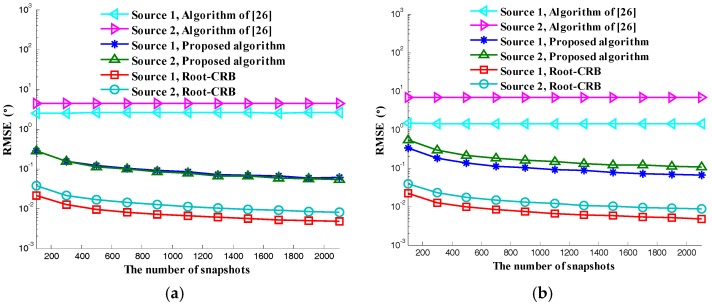

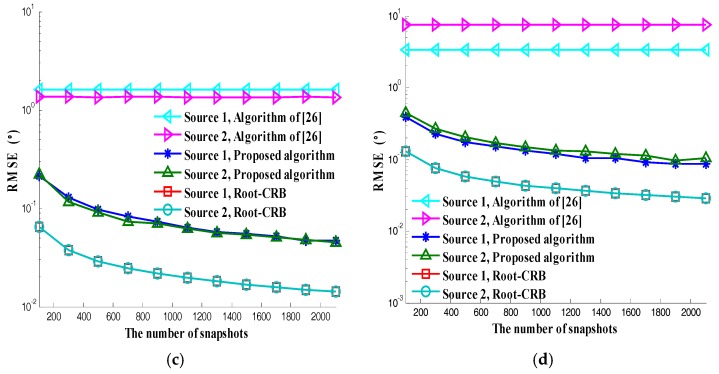
RMSE versus number of snapshots: (**a**) Elevation; (**b**) Azimuth; (**c**) Auxiliary polarization angle; (**d**) Polarization phase difference.

In the third simulation, we compare the RMSE versus the number of snapshots. The number of snapshots is set to vary from 100 to 2100. Other simulation parameters are the same as those of the second simulation. The result is shown in Figure 7. We can see that the C-SPD ESPRIT-like algorithm is getting better and better with the increase of snapshots. There is a gap between the proposed C-SPD ESPRIT-like algorithm and CRB. The reason is same as in the analysis of the second simulation. The performance of the algorithm in [26] is poor no matter how much the snapshots increases.

**Figure 8:**
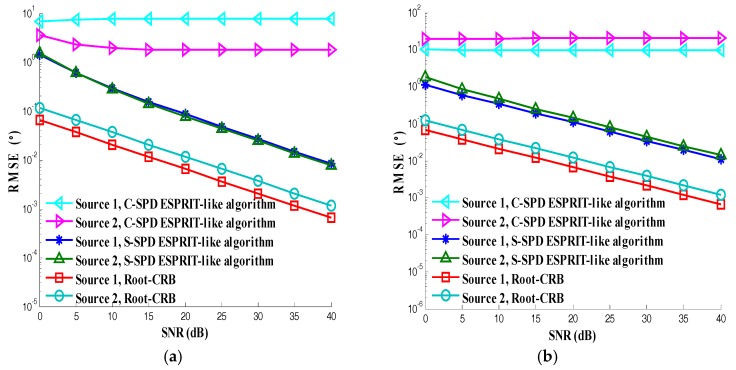

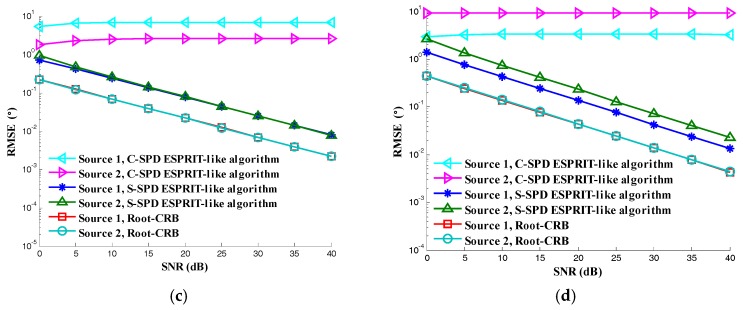
Test of anti mutual coupling: (**a**) Elevation; (**b**) Azimuth; (**c**) Auxiliary polarization angle; (**d**) Polarization phase difference.

In the fourth simulation, we test the estimation accuracy of the S-SPD ESPRIT-like algorithm with separated EMVSs under the condition of GPU and MC. The contrast experiment is the C-SPD ESPRIT-like algorithm with centralized EMVSs under the condition of GPU and MC. The elements of separated EMVSs are set to far enough apart to avoid the MC and the structure is based on the rule we proposed in Equation (45). The mutual coupling matrix of collocated EMVSs is set as a Toeplitz matrix. Other simulation parameters are the same as those of the second simulation. The SNR varies from 0 dB to 40 dB. Two hundred simulations are conducted under each SNR. The simulation results are shown in Figure 8. We can see that the performance of C-SPD ESPRIT-like algorithm is poor no matter how much the signal to noise ratio increases, so the C-SPD ESPRIT-like algorithm with collocated EMVSs can’t deal with the mutual coupling, while we can see that the accuracy of the S-SPD ESPRIT-like algorithm becomes better with the increase of SNR which proves the effectiveness of the S-SPD ESPRIT-like algorithm with separated EMVSs.

## 6. Conclusions

In this work, we research the 2D-DOA and PSA estimation for monostatic MIMO Radar with EMVSs under the condition of GPU and MC. Aiming at resolving the GPU problem, we put forward a C-SPD ESPRIT-like algorithm. The ESPRIT-like algorithm can get good estimation results without knowing the GPU. Furthermore, it is suitable for arbitrary array configurations and has low complexity for avoiding the angle searching procedure. Aiming at the situation where MC and GPU between the elements exist together, we give a class of separated EMVSs and put forward the S-SPD ESPRIT-like algorithm. It can solve the GPU and MC problem simultaneously. Simulation results validate the effectiveness of our algorithms.

## Figures and Tables

**Table 1:** Spatial phase shift factor of separated electromagnetic vector sensor with arbitrary structure.

Single Component Antenna Name	Antenna Position	Spatial Phase Shift Factor
Ex	$\left(x_{ex},y_{ex},z_{ex}\right)$	$d_{ex}=e^{-j\frac{2\pi }{\lambda }\left(x_{ex}u+y_{ex}v+z_{ex}w\right)}$
Ey	$\left(x_{ey},y_{ey},z_{ey}\right)$	$d_{ey}=e^{-j\frac{2\pi }{\lambda }\left(x_{ey}u+y_{ey}v+z_{ey}w\right)}$
Ez	$\left(x_{ez},y_{ez},z_{ez}\right)$	$d_{ez}=e^{-j\frac{2\pi }{\lambda }\left(x_{ez}u+y_{ez}v+z_{ez}w\right)}$
Hx	$\left(x_{hx},y_{hx},z_{hx}\right)$	$d_{hx}=e^{-j\frac{2\pi }{\lambda }\left(x_{hx}u+y_{hx}v+z_{hx}w\right)}$
Hy	$\left(x_{hy},y_{hy},z_{hy}\right)$	$d_{hy}=e^{-j\frac{2\pi }{\lambda }\left(x_{hy}u+y_{hy}v+z_{hy}w\right)}$
Hz	$\left(x_{hz},y_{hz},z_{hz}\right)$	$d_{hz}=e^{-j\frac{2\pi }{\lambda }\left(x_{hz}u+y_{hz}v+z_{hz}w\right)}$

**Table 2:** Comparison of three algorithms.

	Algorithm of Ref. [26]	C-SPD ESPRIT-Like	S-SPD ESPRIT-Like
Anti gain phase uncertainty	N	Y	Y
Anti mutual coupling	N	N	Y
Structure of EMVS	C	C	S
Arbitrary array configuration	Y	Y	Y
Require prior information of target	N	N	Y

‘Y’, ‘N’, ‘C’, ‘S’ represent ‘Yes’, ‘No’, ’Centralized’, ’Separated’ separately.

## Appendix A

Here, we use the method in [28] to derivate the Cramér-Rao Bound (CRB) of our signal model. The parameters need to be estimated are $\left\{\theta_{k},\phi_{k},\gamma_{k},\eta_{k}|k=1,\cdots ,K\right\}$. We can write them as vectors in the following form:

(A1) $$\theta =[\theta_{1},\cdots ,\theta_{K}],\psi =[\phi_{1},\cdots ,\phi_{K}],\gamma =[\gamma_{1},\cdots ,\gamma_{K}],\eta =[\eta_{1},\cdots ,\eta_{K}]$$

Fisher information matrix (FIM) can be represented as

(A2) $$J={\left[\begin{array}{cccc} J_{\theta \theta } & J_{\theta \psi } & J_{\theta \gamma } & J_{\theta \eta }\\ J_{\psi \theta } & J_{\psi \psi } & J_{\psi \gamma } & J_{\psi \eta }\\ J_{\gamma \theta } & J_{\gamma \psi } & J_{\gamma \gamma } & J_{\gamma \eta }\\ J_{\eta \theta } & J_{\eta \psi } & J_{\eta \gamma } & J_{\eta \eta } \end{array}\right]}_{4K\times 4K}$$

On the basis of Ref. [28], the (i,j) elements of $J_{hk},(h,k=\theta ,\psi ,\gamma ,\eta )$ for L snapshots can be written as:

(A3) $$J_{hk}(i,j)=L\cdot \mathrm{Tr}\left\{R^{-1}\frac{\partial R}{\partial h_{i}}R^{-1}\frac{\partial R}{\partial k_{j}}\right\}$$

where R denotes covariance matrix of virtual array. It is assumed that the incident signals are uncorrelated with each other. For convenience, $a_{t}(\theta_{k},\phi_{k})$, $a_{r}(\theta_{k},\phi_{k})$, $a_{\mathrm{pol}}(\theta_{k},\phi_{k},\gamma_{k},\eta_{k})$, and $a(\theta_{k},\phi_{k},\gamma_{k},\eta_{k})$ are abbreviated as $a_{t,k}$, $a_{r,k}$, $a_{\mathrm{pol},k}$, and $a_{k}$, respectively. And the covariance matrix R can be represented as:

(A4) $$R=\sum_{k=1}^{K}\sigma_{s_{k}}^{2}\left\{P_{1}P_{2}[a_{t,k}⊗a_{r,k}⊗a_{\mathrm{pol},k}]\right\}{\left\{P_{1}P_{2}[a_{t,k}⊗a_{r,k}⊗a_{\mathrm{pol},k}]\right\}}^{H}+\sigma_{n}^{2}I_{MN}$$

where $\sigma_{s_{k}}^{2}$ is the power of k incident target. $P_{1}$ and $P_{2}$ are the GPU and MC, respectively. And we compute the first-order partial derivatives of R with regard to $\theta_{k}$, $\phi_{k}$, $\gamma_{k}$ and $\eta_{k}$, as follows:

(A5) $$\frac{\partial R}{\partial \xi_{k}}=\frac{\sigma_{s_{k}}^{2}\partial a_{k}a_{k}^{H}}{\partial \xi_{k}}=\sigma_{s_{k}}^{2}\frac{\partial a_{k}}{\partial \xi_{k}}a_{k}^{H}+\sigma_{s_{k}}^{2}a_{k}\frac{\partial a_{k}^{H}}{\partial \xi_{k}}$$

where ξ denotes θ, ϕ, γ or η. The explicit results of (A5) are shown in the following:

(A6) $${\bar{b}}_{\theta_{k}}\overset{\mathrm{def}}{=}\frac{\partial a_{k}}{\partial \theta_{k}}=P_{1}P_{2}\left\{\begin{array}{l} {}[c_{1}⊙a_{t,k}]⊗a_{r,k}⊗a_{\mathrm{pol},k}+a_{t,k}⊗[c_{2}⊙a_{r,k}]⊗a_{\mathrm{pol},k}\\ +a_{t,k}⊗a_{r,k}⊗\left[c_{9}⊙\left[\Theta (\theta_{k},\phi_{k})g(\gamma_{k},\eta_{k})\right]+d⊙\left[C_{3}g(\gamma_{k},\eta_{k})\right]\right] \end{array}\right\}$$

(A7) $${\bar{b}}_{\phi_{k}}\overset{\mathrm{def}}{=}\frac{\partial a_{k}}{\partial \phi_{k}}=P_{1}P_{2}\left\{\begin{array}{l} {}[c_{4}⊙a_{t,k}]⊗a_{r,k}⊗a_{\mathrm{pol},k}+a_{t,k}⊗[c_{5}⊙a_{r,k}]⊗a_{\mathrm{pol},k}\\ +a_{t,k}⊗a_{r,k}⊗\left[c_{10}⊙\left[\Theta (\theta_{k},\phi_{k})g(\gamma_{k},\eta_{k})\right]+d⊙\left[C_{6}g(\gamma_{k},\eta_{k})\right]\right] \end{array}\right\}$$

(A8) $${\bar{b}}_{\gamma_{k}}\overset{\mathrm{def}}{=}\frac{\partial a_{k}}{\partial \gamma_{k}}=P_{1}P_{2}\left\{a_{t,k}⊗a_{r,k}⊗\left[d⊙[\Theta (\theta_{k},\phi_{k})c_{7}]\right]\right\}$$

(A9) $${\bar{b}}_{\eta_{k}}\overset{\mathrm{def}}{=}\frac{\partial a_{k}}{\partial \eta_{k}}=P_{1}P_{2}\left\{a_{t,k}⊗a_{r,k}⊗\left[d⊙[\Theta (\theta_{k},\phi_{k})c_{8}]\right]\right\}$$

where:

(A10) $$\begin{array}{c} c_{1}=-j\frac{2\pi }{\lambda }[(x_{t1}\cos\theta_{k}\cos\phi_{k}+y_{t1}\cos\theta_{k}\sin\phi_{k}-z_{t1}\sin\theta_{k}),\cdots ,\\ (x_{tM}\cos\theta_{k}\cos\phi_{k}+y_{tM}\cos\theta_{k}\sin\phi_{k}-z_{tM}\sin\theta_{k}){]}^{T} \end{array}$$

(A11) $$\begin{array}{c} c_{2}=-j\frac{2\pi }{\lambda }[(x_{r1}\cos\theta_{k}\cos\phi_{k}+y_{r1}\cos\theta_{k}\sin\phi_{k}-z_{r1}\sin\theta_{k}),\cdots ,\\ (x_{rN}\cos\theta_{k}\cos\phi_{k}+y_{rN}\cos\theta_{k}\sin\phi_{k}-z_{rN}\sin\theta_{k}){]}^{T} \end{array}$$

(A12) $$C_{3}={\left[\begin{array}{llllll} -u_{k}, & -v_{k}, & -w_{k}, & 0, & 0, & 0\\ 0, & 0, & 0, & u_{k}, & v_{k}, & w_{k} \end{array}\right]}^{T}$$

(A13) $$c_{4}=-j\frac{2\pi }{\lambda }{\left[(-x_{t1}v_{k}+y_{t1}u_{k}),\cdots ,(-x_{tM}v_{k}+y_{tM}u_{k})\right]}^{T}$$

(A14) $$c_{5}=-j\frac{2\pi }{\lambda }{\left[(-x_{r1}v_{k}+y_{r1}u_{k}),\cdots ,(-x_{rN}v_{k}+y_{rN}u_{k})\right]}^{T}$$

(A15) $$C_{6}={\left[\begin{array}{llllll} -\cos\theta_{k}\sin\phi_{k}, & \cos\theta_{k}\cos\phi_{k}, & 0, & -\cos\phi_{k}, & -\sin\phi_{k}, & 0\\ -\cos\phi_{k}, & -\sin\phi_{k}, & 0, & \cos\theta_{k}\sin\phi_{k}, & -\cos\theta_{k}\cos\phi_{k}, & 0 \end{array}\right]}^{T}$$

(A16) $$c_{7}={\left[\cos\gamma_{k}e^{j\eta_{k}},-\sin\gamma_{k}\right]}^{T}$$

(A17) $$c_{8}={\left[j\sin\gamma_{k}e^{j\eta_{k}},0\right]}^{T}$$

(A18) $$c_{9}=d⊙\left[\begin{array}{l} -j\frac{2\pi }{\lambda }\left(x_{ex}\cos\theta_{k}\cos\phi_{k}+y_{ex}\cos\theta_{k}\sin\phi_{k}-z_{ex}\sin\theta_{k}\right)\\ -j\frac{2\pi }{\lambda }\left(x_{ey}\cos\theta_{k}\cos\phi_{k}+y_{ey}\cos\theta_{k}\sin\phi_{k}-z_{ey}\sin\theta_{k}\right)\\ -j\frac{2\pi }{\lambda }\left(x_{ez}\cos\theta_{k}\cos\phi_{k}+y_{ez}\cos\theta_{k}\sin\phi_{k}-z_{ez}\sin\theta_{k}\right)\\ -j\frac{2\pi }{\lambda }\left(x_{hx}\cos\theta_{k}\cos\phi_{k}+y_{hx}\cos\theta_{k}\sin\phi_{k}-z_{hx}\sin\theta_{k}\right)\\ -j\frac{2\pi }{\lambda }\left(x_{hy}\cos\theta_{k}\cos\phi_{k}+y_{hy}\cos\theta_{k}\sin\phi_{k}-z_{hy}\sin\theta_{k}\right)\\ -j\frac{2\pi }{\lambda }\left(x_{hz}\cos\theta_{k}\cos\phi_{k}+y_{hz}\cos\theta_{k}\sin\phi_{k}-z_{hz}\sin\theta_{k}\right) \end{array}\right]$$

(A19) $$c_{10}=d⊙\left[\begin{array}{l} -j\frac{2\pi }{\lambda }\left(-x_{ex}v_{k}+y_{ex}u_{k}\right)\\ -j\frac{2\pi }{\lambda }\left(-x_{ey}v_{k}+y_{ey}u_{k}\right)\\ -j\frac{2\pi }{\lambda }\left(-x_{ez}v_{k}+y_{ez}u_{k}\right)\\ -j\frac{2\pi }{\lambda }\left(-x_{hx}v_{k}+y_{hx}u_{k}\right)\\ -j\frac{2\pi }{\lambda }\left(-x_{hy}v_{k}+y_{hy}u_{k}\right)\\ -j\frac{2\pi }{\lambda }\left(-x_{hz}v_{k}+y_{hz}u_{k}\right) \end{array}\right]$$

(A20) $$d=\left[\begin{array}{l} e^{-j\frac{2\pi }{\lambda }\left(-x_{ex}u_{k}+y_{ex}v_{k}+z_{ex}w_{k}\right)}\\ e^{-j\frac{2\pi }{\lambda }\left(-x_{ey}u_{k}+y_{ey}v_{k}+z_{ey}w_{k}\right)}\\ e^{-j\frac{2\pi }{\lambda }\left(-x_{ez}u_{k}+y_{ez}v_{k}+z_{ez}w_{k}\right)}\\ e^{-j\frac{2\pi }{\lambda }\left(-x_{hx}u_{k}+y_{hx}v_{k}+z_{hx}w_{k}\right)}\\ e^{-j\frac{2\pi }{\lambda }\left(-x_{hy}u_{k}+y_{hy}v_{k}+z_{hy}w_{k}\right)}\\ e^{-j\frac{2\pi }{\lambda }\left(-x_{hz}u_{k}+y_{hz}v_{k}+z_{hz}w_{k}\right)} \end{array}\right]$$

Substituting the above results into (A3), the (i,j) elements of FIM $J_{\theta \theta }$ can be expressed as:

(A21) Jθiθj=2Lσsi2σsj2⋅Re{ajHR−1b¯θiaiHR−1b¯θj+ajHR−1aib¯θiHR−1b¯θj}

Define ${\bar{A}}_{\theta }=[{\bar{b}}_{\theta_{1}},{\bar{b}}_{\theta_{2}},\cdots ,{\bar{b}}_{\theta_{K}}]$, then $J_{\theta \theta }$ can be expressed as matrix form:

(A22) Jθθ=2LggT⊙Re{(AHR−1A¯θ)⊙(AHR−1A¯θ)T+(AHR−1A)⊙(A¯θHR−1A¯θ)T}

where $g={\left[\sigma_{s_{1}}^{2},\sigma_{s_{2}}^{2},\cdots ,\sigma_{s_{K}}^{2}\right]}^{T}$. Using the similar derivation, one can obtain:

(A23) Jθψ=JψθT=2LggT⊙Re{(AHR−1A¯ϕ)⊙(AHR−1A¯θ)T+(AHR−1A)⊙(A¯ϕHR−1A¯θ)T}

(A24) Jθγ=JγθT=2LggT⊙Re{(AHR−1A¯γ)⊙(AHR−1A¯θ)T+(AHR−1A)⊙(A¯γHR−1A¯θ)T}

(A25) Jθη=JηθT=2LggT⊙Re{(AHR−1A¯η)⊙(AHR−1A¯θ)T+(AHR−1A)⊙(A¯ηHR−1A¯θ)T}

(A26) Jψψ=2LggT⊙Re{(AHR−1A¯ϕ)⊙(AHR−1A¯ϕ)T+(AHR−1A)⊙(A¯ϕHR−1A¯ϕ)T}

(A27) Jψγ=JγψT=2LggT⊙Re{(AHR−1A¯γ)⊙(AHR−1A¯ϕ)T+(AHR−1A)⊙(A¯γHR−1A¯ϕ)T}

(A28) Jψη=JηψT=2LggT⊙Re{(AHR−1A¯η)⊙(AHR−1A¯ϕ)T+(AHR−1A)⊙(A¯ηHR−1A¯ϕ)T}

(A29) Jγγ=2LggT⊙Re{(AHR−1A¯γ)⊙(AHR−1A¯γ)T+(AHR−1A)⊙(A¯γHR−1A¯γ)T}

(A30) Jγη=JηγT=2LggT⊙Re{(AHR−1A¯η)⊙(AHR−1A¯γ)T+(AHR−1A)⊙(A¯ηHR−1A¯γ)T}

(A31) Jηη=2LggT⊙Re{(AHR−1A¯η)⊙(AHR−1A¯η)T+(AHR−1A)⊙(A¯ηHR−1A¯η)T}

where ${\bar{A}}_{\phi }=[{\bar{b}}_{\phi_{1}},{\bar{b}}_{\phi_{2}},\cdots ,{\bar{b}}_{\phi_{K}}]$, ${\bar{A}}_{\gamma }=[{\bar{b}}_{\gamma_{1}},{\bar{b}}_{\gamma_{2}},\cdots ,{\bar{b}}_{\gamma_{K}}]$, and ${\bar{A}}_{\eta }=[{\bar{b}}_{\eta_{1}},{\bar{b}}_{\eta_{2}},\cdots ,{\bar{b}}_{\eta_{K}}]$. Now, the FIM J has been obtained and any unbiased estimation would have these Cramér-Rao lower bounds:

(A32) $$\left\{ \begin{array}{l} \mathrm{CRB}(\theta_{k})={\left[J^{-1}\right]}_{k,k}\\ \mathrm{CRB}(\phi_{k})={\left[J^{-1}\right]}_{K+k,K+k}\\ \mathrm{CRB}(\gamma_{k})={\left[J^{-1}\right]}_{2K+k,2K+k}\\ \mathrm{CRB}(\eta_{k})={\left[J^{-1}\right]}_{3K+k,3K+k} \end{array}\right.$$
